# *In Site* Bioimaging of Hydrogen Sulfide Uncovers Its Pivotal Role in Regulating Nitric Oxide-Induced Lateral Root Formation

**DOI:** 10.1371/journal.pone.0090340

**Published:** 2014-02-27

**Authors:** Yan-Jun Li, Jian Chen, Ming Xian, Li-Gang Zhou, Fengxiang X. Han, Li-Jun Gan, Zhi-Qi Shi

**Affiliations:** 1 College of Life Sciences, Nanjing Agricultural University, Nanjing, China; 2 Institute of Food Quality and Safety, Jiangsu Academy of Agricultural Sciences, Nanjing, China; 3 Department of Chemistry, Washington State University, Pullman, Washington, United States of America; 4 Department of Plant Pathology, College of Agronomy and Biotechnology, China Agricultural University, Beijing, China; 5 Department of Chemistry and Biochemistry, Jackson State University, Jackson, Mississippi, United States of America; Juntendo University School of Medicine, Japan

## Abstract

Hydrogen sulfide (H_2_S) is an important gasotransmitter in mammals. Despite physiological changes induced by exogenous H_2_S donor NaHS to plants, whether and how H_2_S works as a true cellular signal in plants need to be examined. A self-developed specific fluorescent probe (WSP-1) was applied to track endogenous H_2_S in tomato (*Solanum lycopersicum*) roots in site. Bioimaging combined with pharmacological and biochemical approaches were used to investigate the cross-talk among H_2_S, nitric oxide (NO), and Ca^2+^ in regulating lateral root formation. Endogenous H_2_S accumulation was clearly associated with primordium initiation and lateral root emergence. NO donor SNP stimulated the generation of endogenous H_2_S and the expression of the gene coding for the enzyme responsible for endogenous H_2_S synthesis. Scavenging H_2_S or inhibiting H_2_S synthesis partially blocked SNP-induced lateral root formation and the expression of lateral root-related genes. The stimulatory effect of SNP on Ca^2+^ accumulation and *CaM1* (*calmodulin 1*) expression could be abolished by inhibiting H_2_S synthesis. Ca^2+^ chelator or Ca^2+^ channel blocker attenuated NaHS-induced lateral root formation. Our study confirmed the role of H_2_S as a cellular signal in plants being a mediator between NO and Ca^2+^ in regulating lateral root formation.

## Introduction

Hydrogen sulfide (H_2_S) is considered as the third gasotransmitter in medical biology after nitric oxide (NO) and carbon monoxide (CO) [1]. The clinical relevance of H_2_S as a signaling molecule has been highly appreciated in mammals [2]–[4]. In mammals and bacteria, two multifunctional pyridoxal 5′-phosphate (PLP)-dependent enzymes, cystathionine γ-lyase (CSE) and cystathionine β-synthase (CBS), are demonstrated to be the major sources of endogenous H_2_S production [5]. H_2_S can also be produced by 3-mercaptopyruvate sulfurtransferase (3SMT) along with cysteine aminotransferase (CAT) in brain [6]. In plants H_2_S is considered to be a by-product from cysteine desulfuration catalyzed by _L_-cysteine desulfhydrase (LCD, EC4.4.1.1) and _D_-cysteine desulfhydrase (DCD, EC4.4.1.15), both of which belonging to the PLP protein family [7]. Both genes (*LCD* and *DCD*) have been characterized in *Arabidopsis* [8]. A recent study suggests that *O*-acetylserine(thiol)lyase (OASTL), a cysteine synthase-like protein, also possesses the activity of cysteine desulfuration [9].

The detailed studies in the biological role of H_2_S in plants are very limited compared to those in mammals [10]. Exogenous application of NaHS, a H_2_S donor, confers the tolerance of plants to oxidative stress [11]–[20]. H_2_S is also proposed to be involved in regulating stomatal closure [21]–[23], photosynthesis [24], and seed germination [25], [26]. However, the major challenge of identifying the nature of H_2_S as a plant signaling molecule is the lack of data of tracking endogenous H_2_S in site in plants. The traditional approaches of determining H_2_S from biological tissues include colorimetric in-tube assay [27], sulfide electrode assay [21], and gas chromatography/mass spectrometry [28]. These methods require tissue pre-processing (e.g. homogenization), leading to the unavoidable loss of H_2_S. Therefore, in the last two years, a group of chemists have developed some specific fluorescent probes for capturing and tracking H_2_S *in vivo* through instantaneous bioimaging [29], which show great potential for revealing the biological behavior of H_2_S. However, the application of these probes in biological study, especially for plants, is rarely reported.

NO-modulated lateral root formation is a well characterized signaling event in plants [30], [31]. NO can modulate the expression of cell cycle regulatory genes (e.g. *CYCD* and *CDKA*), which are essential for lateral root initiation from primordia [32], [33]. The key of lateral root formation is lateral root emergence, which is a process that new primordia break through the outer layer cells from primary roots [33]. Auxin has been confirmed as a regulatory star in this process by positively regulating Auxin Response Factors (ARFs) (e.g. ARF4/7/19) [33]–[35] and endogenous NO [30]. In addition, a recent study suggests that cytosolic Ca^2+^ combined with its sensor calmodulin (CaM) acts downstream of NO during lateral root formation [36]. The auxin-NO signaling event has been considered to play a vital role in regulating lateral root growth, but the detailed regulatory network needs to be illuminated by mining novel components. The biological interplay among H_2_S, NO, and Ca^2+^ has been well investigated in mammals [37], [38]. Thus, it is of interest to study whether and how H_2_S acts as a gasotransmitter in NO signaling cassette for the regulation of lateral root formation. WSP-1 (Washington State Probe-1) is a self-developed fluorescent probe for detecting H_2_S within living cells with high-sensitivity and selectivity [39], [40]. In the present study, tracking and bioimaging endogenous H_2_S with WSP-1 in plant cells provide direct evidence that H_2_S is a novel regulator in NO-modulated lateral root formation. This study confirmed the role of H_2_S as a cellular signal molecule in plant signaling events.

## Materials and Methods

### Plant culture and treatments

Tomato (*Solanum lycopersicum*, Suhong2003 wild type) seeds were surface-sterilized with 1% NaClO for 10 min followed by washing with distilled water. Seeds were germinated in Petri dishes on filter papers imbibed with distilled water. Then the selected identical seedlings with radicles 1.5 cm were transferred to another Petri dish containing various treatment solutions in a chamber with a photosynthetic active radiation of 200 μmol/m^2^/s, a photoperiod of 12 h, and the temperature at 25±1°C.

SNP (sodium nitroprusside) and GSNO (S-Nitrosoglutathione) as NO donors were applied at concentrations of 0.05–0.4 mM and 0.5 mM, respectively. The 0.1 mM of cPTIO [2-(4-carboxy-2-phenyl)-4,4,5,5-tetramethylinidazoline-1-oxyl-3-oxide] was applied as NO scavenger. The 0.2–2 mM of NaHS (sodium hydrosulphide) was applied as H_2_S donor. PAG (_DL_-propargylglicine) (0.1 mM) and HT (hypotaurine) (0.1 mM) are H_2_S biosynthesis inhibitors and H_2_S scavengers, respectively. Na_2_SO_4_, Na_2_SO_3_, and NaHSO_3_ at the concentration of 2 mM are applied as NaHS homologues to identify the specificity for NaHS as H_2_S donor. EGTA [ethylene glycol-bis(2-aminoethylether)-N,N,N,N-tetraacetic acid] (0.1 mM) and LaCL_3_ (0.5 mM) are Ca^2+^ chelators and Ca^2+^ channel blockers, respectively. The treatment solution is composed of different chemicals as mentioned above according to the experimental design. After treatments, the roots were washed with distilled water for physiological, histochemical, and biochemical analysis.

### Histochemical detection of endogenous H_2_S and cytosolic Ca^2+^ *in vivo*

Intracellular NO was visualized using DAF-FM DA (3-Amino, 4-aminomethyl-2′,7′- difluorescein, diacetate) fluorescent probe described by Guo et al [41]. The roots of seedlings after treatment were transferred to 20 mM of Hepes-NaOH (pH 7.5) buffer solution containing 15 μM of DAF-FM DA. After being incubated in darkness at 25°C for 15 min, the roots were rinsed with distilled water for three times and were visualized (excitation 490 nm and emission 525 nm) by a fluorescence microscope (ECLIPSE, TE2000-S, Nikon).

The intracellular H_2_S was visualized using WSP-1 [3′- methoxy- 3- oxo- 3H- spiro[isobenzofuran- 1, 9′- xanthen]- 6′- yl 2- (pyridin- 2- yldisulfanyl)benzoate]. The roots of seedlings after treatments were transferred to 20 mM Hepes-NaOH (pH 7.5) buffer solution containing 15 μM of WSP-1. After being incubated in darkness at 25°C for 40 min, the roots were washed with distilled water three times and were visualized immediately by a fluorescence microscope with a 465/515 nm and an excitation/emission filter set (ECLIPSE, TE2000-S, Nikon).

For the detection of WSP-1 fluorescence in different reactive sulfur species, WSP-1 with final concentration of 15 μM were added into the following solutions, SDS (sodium dodecyl sulfate, 2 mM), NaHSO_4_ (2 mM), NaHSO_3_ (2 mM), Na_2_SO_4_ (2 mM), Na_2_SO_3_ (2 mM), Na_2_S_2_O_4_ (2 mM), GSSG (glutathione disulfide, 2 mM), sulfonamide (2 mM), GSNO (2 mM), and NaHS (2 mM), respectively. Twenty μL of the above solutions were transferred to a glass slide for the visualization with a fluorescence microscope with a 465/515 nm and an excitation/emission filter set (ECLIPSE, TE2000-S, Nikon).

The cytosolic Ca^2+^ was visualized using Ca^2+^-sensitive fluorescent probe Fluo-3 AM. Similarly, the probe was loaded to roots in 20 mM Hepes-NaOH (pH 7.5) buffer solution containing 15 μM of Fluo-3 AM in darkness at 25°C for 30 min. Then, the fluorescent image was captured using a fluorescence microscope with 488/525 nm and an excitation/emission filter set (ECLIPSE, TE2000-S, Nikon).

The relative fluorescent density of the fluorescent images was analyzed using Image-Pro Plus 6.0 (Media Cybernetics, Inc.).

### Analysis of transcripts

Semiquantitative RT-PCR was performed with the total RNA for the transcription analysis. Total RNA was extracted from root samples using Trizol (Invitrogen) according to the manufacturer's instructions. Reverse transcription was performed at 42°C in a 25 μl reaction mixture including 3 μg of RNA, 0.5 μg of oligo(dT) primers, 12.5 nmol of dNTPs, 20 units of RNase inhibitor and 200 units of MLV. The first cDNA was used as a template for PCR to analyze the transcripts of genes. The total 25 μl of PCR reaction mixture in Tris-HCl buffer (pH 8.3, 10 mM) was composed of 1 μl of normalized cDNA template, 10 pmol of sense primer, 10 pmol of antisense primer, 5 nmol of dNTPs, 32.5 nmol of Mg^2+^, and 0.5 U of Tag DNA polymerase. PCR was performed as follows: 95°C for 3 min, 30 cycles at 94°C for 30 s, different annealing temperature for 30 s, 68°C for 1.5 min, and a final extension step at 68°C for 7 min. All the tested genes were retrieved from tomato genome (Sol Genomics Network, http://solgenomics.net/organism/Solanum_lycopersicum/genome) or NCBI (National Center for Biotechnology Information, http://www.ncbi.nlm.nih.gov/). The following primers and annealing temperatures were used to amplify the genes: *CYCD3;1* (Sol accession number SGN-U583476), sense 5′-TTATCTTTCATTGATCATATTATGAGG-3′ and antisense 5′-CTAGGTAATCTAGAGAACAAGATATCG-3′ (amplifying a 526 bp fragment, 45°C); *CDKA1* (Sol accession number SGN-U572518), sense 5′-GCTTATTGTCATTCTCATAGAGTTCTT-3′ and antisense 5′-TCGTTGAAGCACTCATGCTCAAGGGC-3′ (Sol amplifying a 521 bp fragment, 45°C); *ARF4* (Sol accession number SGN-U569639), sense 5′-ATGCTTGTGCTGGTCC-3′ and antisense 5′-CTCCGTGCAGATCCTT-3′ (amplifying a 477 bp fragment, 45°C); *ARF7* (NCBI accession number EF121545.1), sense 5′-TCAGAGTTATGGCACG-3′ and antisense 5′-GACGAGGAACAGAAAA-3′ (amplifying a 373 bp fragment, 45°C); *CaM1* (Sol accession number SGN-U580544), sense 5′-TGAATCTGATGGCACGGAAG-3′ and antisense 5′-TACTTGAACCGCTCCTGAGT-3′ (amplifying a 338 bp fragment, 50°C); *Actin* (Sol accession number SGN-U580422), sense 5′-AGAGCTATGAGCTCCCAGATGG-3′ and antisense 5′-TTAATCTTCATGCTGCTAGGAGC-3′ (amplifying a 272 bp fragment, 50°C). The relative abundance of *Actin* was used as internal standard.

### Statistical analysis

Each result was presented as the mean of at least three replicated measurements. The significant differences between treatments were statistically evaluated by standard deviation and one-way analysis of variance (ANOVA) using Microsoft Excel 2010 (Microsoft Corporation, USA). The data between different treatments were compared statistically by ANOVA, followed by F-test if the ANOVA result is significant at *P*<0.05.

## Results

### NO induced lateral root formation

**Figure 1:**
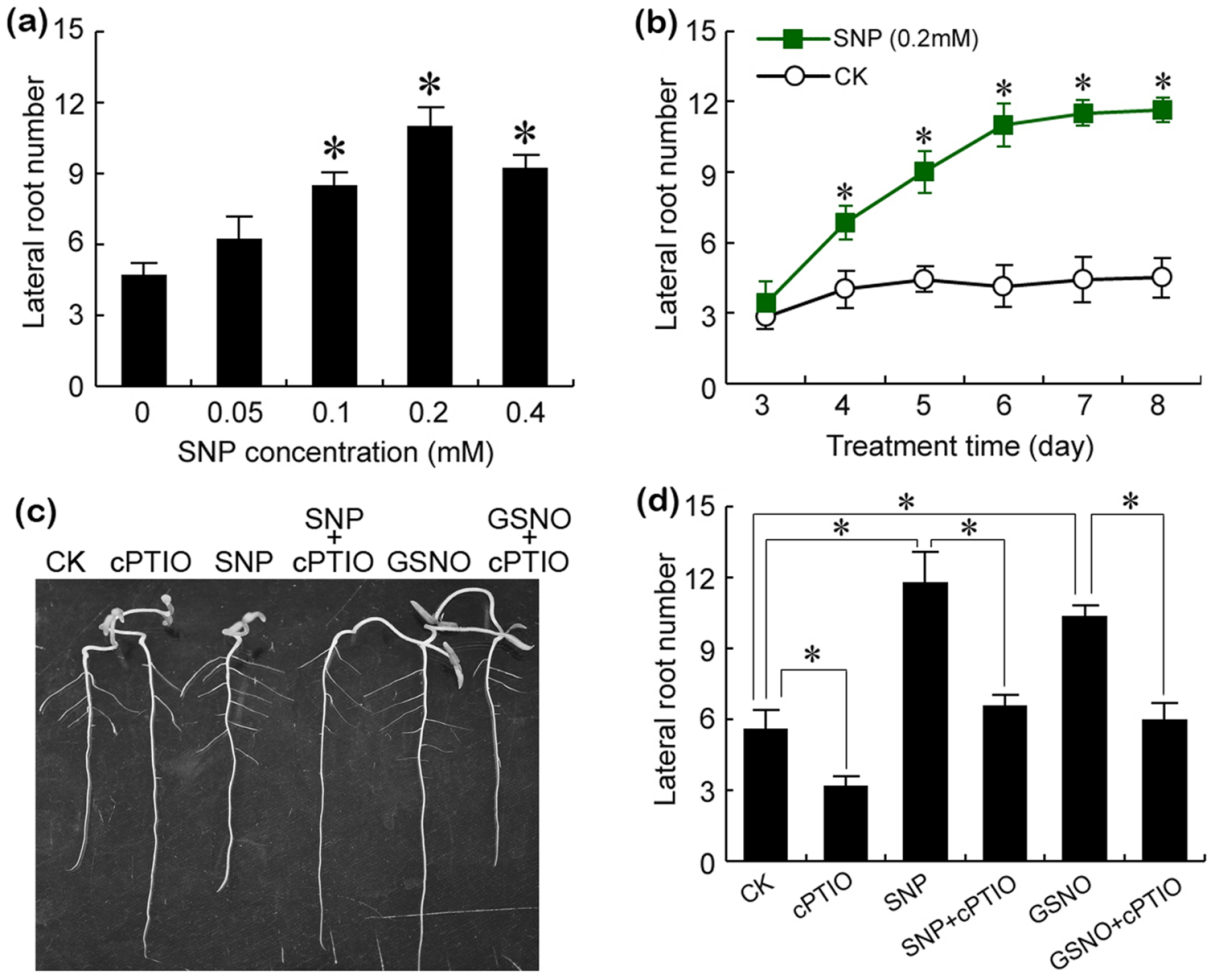
NO induced lateral root formation in both dose- and time-dependent manner. (a) The roots of three-day old tomato seedlings were exposed to 0, 0.05, 0.1, 0.2, and 0.4 mM of SNP for 6 days for the measurement of the lateral root number. (b) The roots of three-day old tomato seedlings were exposed to 0.2 mM of SNP for 3–8 days for the measurement of the lateral root number. (c–d) The roots of three-day old tomato seedlings were exposed to cPTIO (0.1 mM), SNP (0.2 mM), SNP (0.2 mM) + cPTIO (0.1 mM), GSNO (0.5 mM), and GSNO (0.5 mM) + cPTIO (0.1 mM) for 6 days for photographing root phenotype (c) and measuring the lateral root number (d). Vertical bars represent standard deviations of the mean (n = 6). Asterisk indicates that mean values are significantly different (*P*<0.05) between the treatment and the control (a, b) or between different treatments (d).

NO donors were used to assess the regulatory effect of NO on tomato lateral root formation. The NO donor SNP stimulated lateral root growth in both dose- and time-dependent manner (Figure 1a and b). On the contrary, treatments with NO scavenger cPTIO alone remarkably inhibited lateral root formation (Figure 1c and d). Another NO donor GSNO could stimulate lateral root formation as well (Figure 1c and d). However, the addition of cPTIO could abolish the promoting effect of both NO donors on lateral roots(Figure 1c and d). These results confirmed the promoting effect of NO on tomato lateral root formation.

### WSP-1 can be used for the selective detection of H_2_S in tomato root

**Figure 2:**
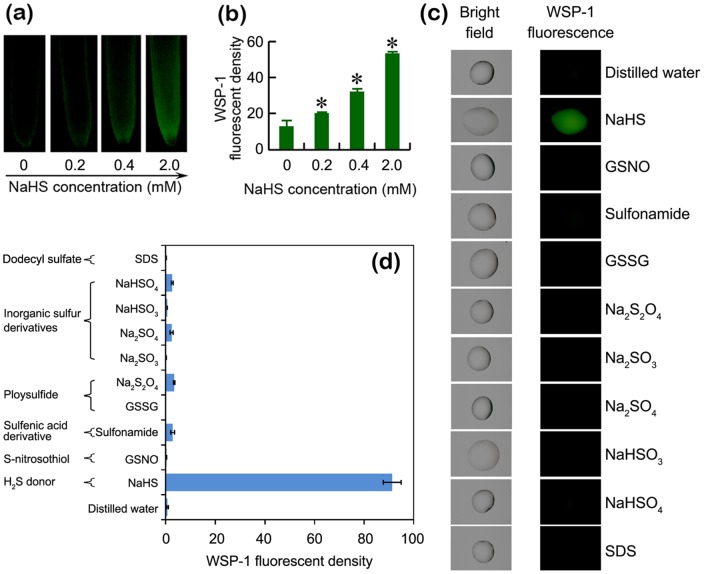
WSP-1 was a selective fluorescent probe for detecting H_2_S in tomato roots. (a–b) The roots of three-day old tomato seedlings were exposed to NaHS solution (0, 0.2, 0.4, and 2 mM) for 2 h. Then the roots were loaded with WSP-1 for fluorescent imaging (a) and the calculation of relative fluorescent density (b). (c) WSP-1 was loaded into different sulfur compounds solutions as mentioned in “Materials and Methods” for fluorescent imaging. (d) The calculation of relative fluorescent density from (c). Vertical bars represent standard deviations of the mean (n = 3). Asterisk indicates that mean values are significantly different (*P*<0.05) between the treatment and the control.

In order to investigate the potential of WSP-1 in the detection of H_2_S in plant system, tomato roots treated with NaHS at different concentrations (0.2, 0.4, and 2 mM) were loaded with WSP-1. These concentrations were within the range of those that have been used to elicit physiological responses of H_2_S in plants [19], [20], [24], [42]. The strong fluorescent density was observed in roots in the presence of NaHS in a dose-dependent manner (Figure 2a and b). This result was similar to the detection of H_2_S with WSP-1 in mammalian system [40]. To further identify the selectivity of WSP-1 probe for H_2_S, several kinds of reactive sulfur species (e.g. sulfane sulfur, inorganic sulfur derivatives, ploysulfide, sulfenic acid derivative, and S-nitrosothiol) were detected in solution. As expected, compared to the significant fluorescence signal yielded from the reaction of WSP-1 with NaHS solution, other tested reactive sulfur species did not lead to significant fluorescence increase (Figure 2c). Analysis of fluorescent density showed that several sulfur compounds (e.g. NaHSO_4_, Na_2_SO_4_, Na_2_S_2_O_4_, and sulfonamide) had little fluorescence, but their values are too small as compared with NaHS (Figure 2d). These results suggested that WSP-1 could be used for the selective detection of endogenous H_2_S in tomato roots.

### Endogenous H_2_S was involved in lateral root formation

**Figure 3:**
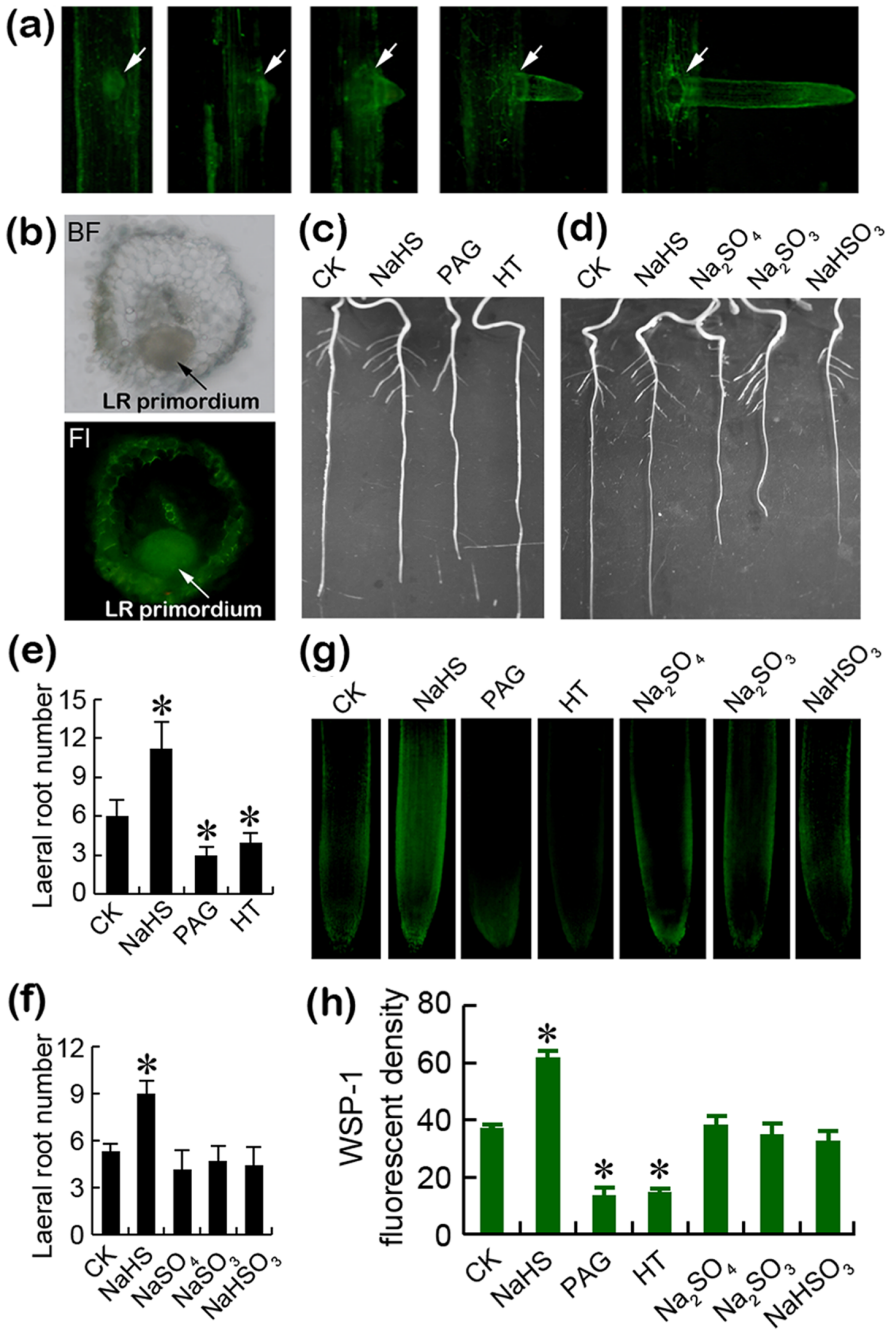
Endogenous H_2_S was involved in lateral root formation in tomato seedlings. (a) WSP-1 was loaded into the roots during lateral root formation for fluorescent imaging. (b) The cross section of primary roots loaded with WSP-1. Arrows indicate lateral roots (LR) primordium; BF, bright field; Fl, fluorescence. (c–f) The roots of three-day old tomato seedlings were exposed to NaHS (2 mM), PAG (0.1 mM), HT (0.1 mM), Na_2_SO_4_ (2 mM), Na_2_SO_3_ (2 mM), and NaHSO_3_ (2 mM) for 6 days for photographing root phenotype (c–d) and measuring lateral root numbers (e–f). Vertical bars represent the standard deviations of the mean (n = 6). (g–h) The roots of three-day old tomato seedlings were exposed to NaHS (2 mM), PAG (0.1 mM), HT (0.1 mM), Na_2_SO_4_ (2 mM), Na_2_SO_3_ (2 mM), and NaHSO_3_ (2 mM) for 3 days. Then, the roots were loaded with WSP-1 for fluorescent imaging (g) and the calculation of relative fluorescent density (h). Vertical bars represent standard deviations of the mean (n = 3). Asterisk indicates that mean values are significantly different (*P*<0.05) between the treatment and the control.

Next, we investigate the link between lateral root emergency and endogenous H_2_S. The bright green fluorescence of WSP-1 was clearly linked to the primordium initiation and lateral root emergence (Figure 3a). In a cross section of primary roots with lateral root primoidium, the fluorescence of WSP-1 was clearly concentrated in the region of primordium (Figure 3b). To further ascertain the role of H_2_S in regulating lateral root formation, we measured lateral root number by altering endogenous H_2_S level in roots. Both H_2_S biosynthesis inhibitor PAG and H_2_S scavenger HT induced significant decreases in lateral root number (Figure 3c and e). However, the treatment with NaHS significantly enhanced lateral root number compared to the control (Figure 3c and e). Treatments with several homologues of Na or S (e.g. Na_2_SO_4_, Na_2_SO_3_, and NaHSO_3_) did not affect lateral root number (Figure 3d and f), suggesting that NaHS-released H_2_S contributed to the promotion of lateral root formation. This was further confirmed by *in vivo* fluorescent detection of endogenous H_2_S level in roots. Both PAG and HT led to the significant decrease in endogenous H_2_S levels (Figure 3g and h). NaHS, but not its homologues, remarkably enhanced endogenous H_2_S level in roots (Figure 3g and h).

### NO induced lateral root formation by regulating endogenous H_2_S generation

**Figure 4:**
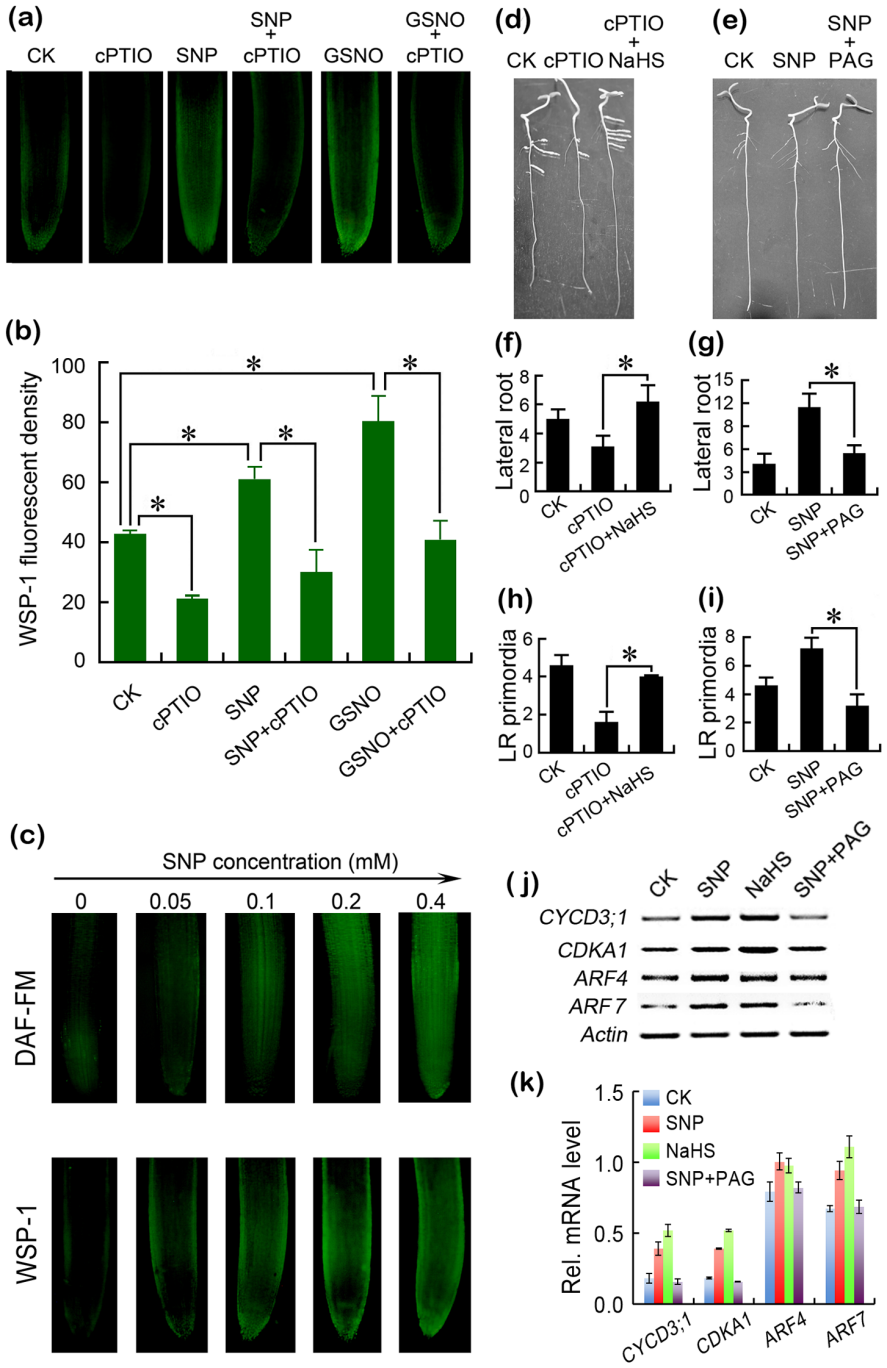
NO induced lateral root formation by regulating endogenous H_2_S generation. (a–b) The roots of three-day old tomato seedlings were exposed to cPTIO (0.1 mM), SNP (0.2 mM), SNP (0.2 mM)+cPTIO (0.1 mM), GSNO (0.5 mM), and GSNO (0.5 mM)+cPTIO (0.1 mM) for 3 days. Then, the roots were loaded with WSP-1 for fluorescent imaging (a) and the calculation of relative fluorescent density (b). Vertical bars represent standard deviations of the mean (n = 3). (c) The roots of three-day old tomato seedlings were exposed to 0, 0.05, 0.1, 0.2, and 0.4 mM of SNP for 3 days. Then, the roots were loaded with DAF-FM DA and WSP-1 for fluorescent imaging, respectively. (d–g) The roots of three-day old tomato seedlings were exposed to cPTIO (0.1 mM), cPTIO (0.1 mM) + NaHS (2 mM), SNP (0.2 mM), and SNP (0.2 mM) + PAG (0.1 mM) for 6 days for photographing root phenotype (d–e) and measuring lateral root number (f–g). (h–i) The roots of three-day old tomato seedlings were exposed to cPTIO (0.1 mM), cPTIO (0.1 mM) + NaHS (2 mM), SNP (0.2 mM), and SNP (0.2 mM) + PAG (0.1 mM) for 2 days for the measurement of lateral root primordia. Vertical bars represent standard deviations of the mean (n = 6). (j) The roots of three-day old tomato seedlings were exposed to SNP (0.2 mM), NaHS (2 mM), and SNP (0.2 mM)+PAG (0.1 mM) for 2 days for the analysis of genes transcripts. (k) Quantitative analysis of genes transcript levels under different treatment conditions. The data were obtained by densitometric analysis of the relative abundance of the transcripts with respect to the loading control *Actin*. Asterisk indicates that mean values are significantly different (*P*<0.05) between the treatment and the control. *Actin* was used for cDNA normalization.

To determine the role of H_2_S in NO-induced lateral root formation, we first investigated the effect of NO donors on the generation of endogenous H_2_S in roots detected by WSP-1. Treatments with NO scavenger cPTIO induced a significant decrease in endogenous H_2_S level in roots (Figure 4a and b). Two NO donors (SNP and GSNO) stimulated the generation of endogenous H_2_S, which could be blocked by the addition of cPTIO (Figure 4a and b). SNP stimulated the generation of endogenous NO and H_2_S in dose-dependent manners (Figure 4c).

Since NO was able to stimulate H_2_S generation in tomato roots, it is essential to know whether NO-governed H_2_S generation is able to manipulate lateral root formation. The addition of NaHS reversed the inhibitory effect of cPTIO on lateral root formation (Figure 4d and f). Furthermore, the addition of PAG abolished the stimulatory effect of SNP on lateral root formation (Figure 4e and g). These effects could be observed in the emergence of lateral root primordia as well (Figure 4h and i). Then we tested the effect of the interplay between NO and H_2_S on the expression of four genes related to lateral root emergence, including two cell cycle regulatory genes (*CYCD3;1* and *CDKA1*) and two ARF genes (*ARF4* and *ARF7*). As expected, both SNP and NaHS could stimulate the expression of these genes while the addition of PAG could block the stimulatory effect of SNP (Figure 4j and k).

### Ca^2+^/*CaM1* acted downstream of H_2_S in NO-induced lateral root formation

**Figure 5:**
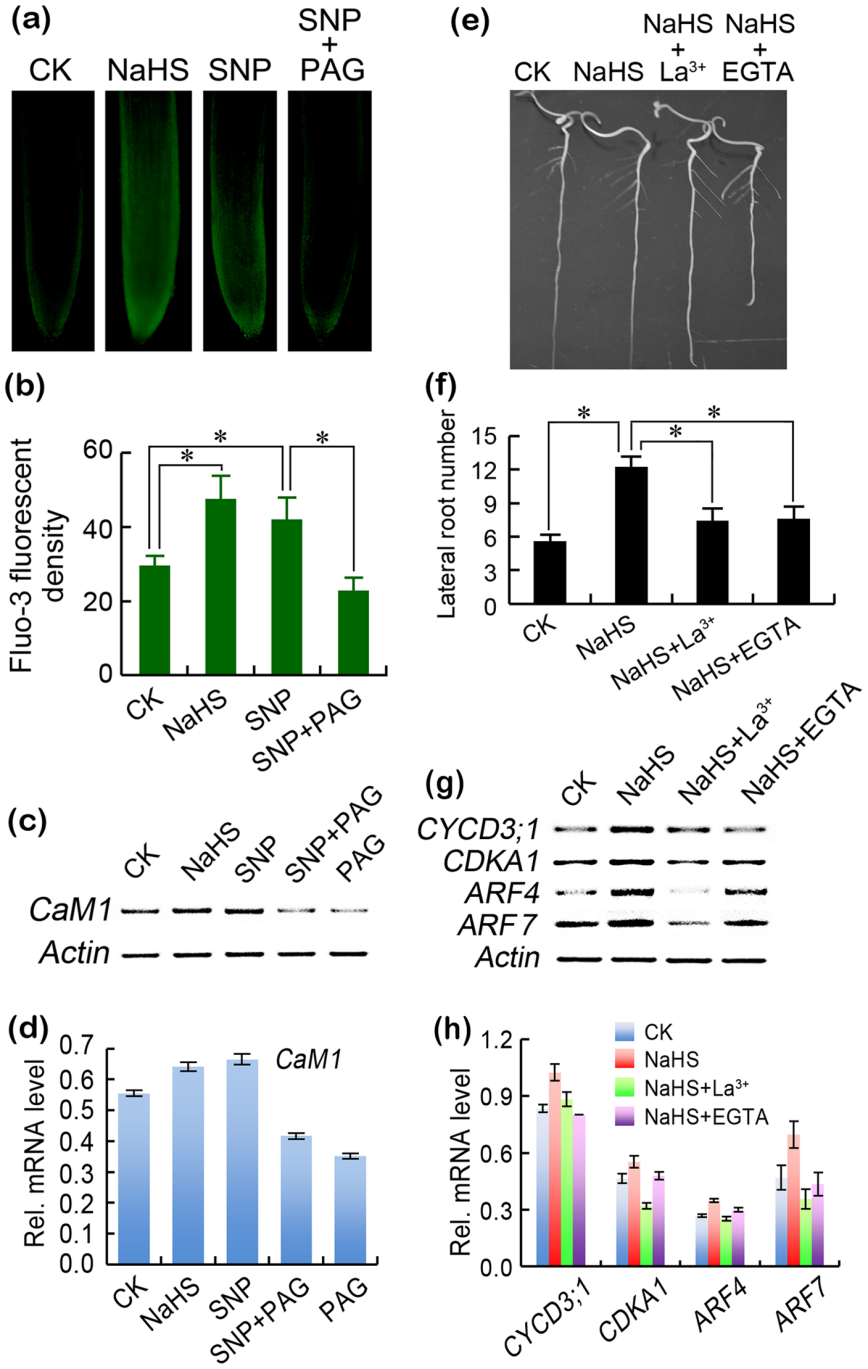
Ca^2+^ acted downstream of H_2_S in the NO-induced lateral root formation. (a–c) The roots of three-day old tomato seedlings were exposed to SNP (0.2 mM), NaHS (2 mM), and SNP (0.2 mM) + PAG (0.1 mM) for 4 days. Then, the roots were loaded with Fluo-3 AM for fluorescent imaging (a) and the calculation of relative fluorescent density (b). Vertical bars represent standard deviations of the mean (n = 3). The roots were also used for the analysis of the *CaM1* transcripts (c). (d) Quantitative analysis of *CaM1* transcript levels under different treatment conditions. The data were obtained by densitometric analysis of the relative abundance of the transcripts with respect to the loading control *Actin*. (e–f) The roots of three-day old tomato seedlings were exposed to NaHS (2 mM), NaHS (2 mM)+La^3+^ (0.5 mM), and NaHS (2 mM)+EGTA (0.1 mM) for 6 days for photographing root phenotype (e) and measuring lateral root numbers (f). Vertical bars represent standard deviations of the mean (n = 6). (g) The roots of three-day old tomato seedlings were exposed to NaHS (2 mM), NaHS (2 mM)+La^3+^ (0.5 mM), and NaHS (2 mM)+EGTA (0.1 mM) for 2 days for the analysis of genes transcripts. (h) Quantitative of genes transcript levels under different treatment conditions. The data were obtained by densitometric analysis of of the relative abundance of the transcripts with respect to the loading control *Actin*. Asterisk indicates that mean values are significantly different (*P*<0.05) between different treatments. *Actin* was used for cDNA normalization.

By using Fluo-3 AM to detect cytosolic Ca^2+^ in tomato roots, we found that both SNP and NaHS stimulated the accumulation of cytosolic Ca^2+^ in roots while the addition of PAG reversed the stimulatory effect of SNP and NaHS (Figure 5a and b). The changes in the *CaM1* expression showed similar patterns with cytosolic Ca^2+^ under the above treatments (Figure 5c and d).

Next, we determined the cross-talk between H_2_S and Ca^2+^ on lateral root formation. As expected, both Ca^2+^ channel blocker La^3+^ and Ca^2+^ chelator EGTA could abolish the stimulatory effect of NaHS on lateral root formation (Figure 5e and f) and the expression of *CYCD3;1*, *CDKA1*, *ARF4*, and *ARF7* (Figure 5g and h).

## Discussion

In plants, both NO and CO have been already identified as vital signaling molecules participating in an array of intrinsic signaling events [43], [44]. But the biology of H_2_S in plants is not well understood. Many physiological changes in plants resulting from the exposure of exogenous H_2_S have been summarized by Lisjak et al. [10]. In order to identify whether H_2_S is a true cellular signal in plants, the in site concentration and the locality of endogenous H_2_S in plants need to be determined [10]. In the present study, we overcome the obstacle of tracking endogenous H_2_S in plants in site. The endogenous H_2_S in tomato roots have been successfully detected in site by specific fluorescent probe WSP-1, which provides the direct evidence supporting the interplay among NO, H_2_S, and Ca^2+^ in regulating lateral root formation.

Basically, we provide three lines of evidence indicating that H_2_S is an important messenger operating downstream of NO during lateral root formation in tomato seedlings. First, endogenous H_2_S accumulation accompanies with lateral root emergency while lateral root formation is positively correlated with the changes of endogenous H_2_S concentration in pharmacological experiments. Second, PAG- or HT-induced decreases in endogenous H_2_S blocked NO-induced lateral root formation. Third, PAG is able to attenuate the stimulatory effect of NO on cytosolic Ca^2+^ accumulation and *CaM1* transcripts. Both Ca^2+^ channel blocker and Ca^2+^ chelator can inhibit H_2_S-induced lateral root formation.

The development of specific fluorescent probes provides a powerful tool for studying the biological function of gasotransmitters. WSP-1 has been demonstrated to be efficient fluorescence probe for selectively detecting H_2_S in mammalian system [40]. Whether WSP-1 is suitable for the detection of H_2_S in plants remains unclear because of the abundant reactive sulfur species in plants [45]. In the present study, NaHS, but not other five kinds of tested reactive sulfur species, can react with WSP-1 to produce significant fluorescence *in vitro*. Tomato roots treated with NaHS showed well-increased fluorescence detected by WSP-1. These evidences support that WSP-1 shows great potential for selectively detecting H_2_S in tomato roots. Our study widens the application of WSP-1 for detecting H_2_S in biological system.

According to the detection of endogenous NO using famous fluorescent probe DAF-2 DA, NO accumulation is clearly associated to lateral root primodium initiation [30]. Interestingly, the link between endogenous H_2_S accumulation and primodium emergence has been established successfully with using specific fluorescent probe WSP-1 in the present study. Based on our data, the endogenously generated NO induced lateral root formation through endogenous H_2_S generation. This can be confirmed by the fact that the decreases in the concentration of endogenous NO and H_2_S caused the inhibition of lateral root formation while treatment with NO donor SNP enhanced the concentration of endogenous NO, resulting in the increase in the concentration of endogenous H_2_S in roots. The promoting effect of NO on lateral root formation has been well characterized by Correa-Aragunde et al [30]. Here, we demonstrate that H_2_S is a new component of the signaling event for NO-induced lateral root formation, and that H_2_S acts downstream of NO signal. But Zhang et al. has reported that H_2_S may act upstream of NO in inducing adventitious root formation. However, a possible feedback regulation of H_2_S by NO has also been suggested because plant roots treated with SNP maintained higher levels of endogenous H_2_S in comparison to control [46]. Our present study provides the detailed evidence that NO induces lateral root formation by regulating endogenous H_2_S. Therefore, it can be proposed that H_2_S is required for root organogenesis by functioning probably both upstream and downstream of NO. However, whether and how NO induces H_2_S generation by regulating LCD or DCD in tomato roots remains to be further investigated.

The function of Ca^2+^ as a mediator in NO-induced lateral root formation of *Arabidopsis* has recently been studied by Wang et al [36]. In tobacco suspension cultured cells, the application of Ca^2+^ chelator or CaM antagonists can decrease NaHS-induced heat tolerance, supporting that Ca^2+^ may act downstream of H_2_S [13]. Here we suggests that H_2_S is a mediator between NO and Ca^2+^ in lateral root development of tomato plants by fluorescently bioimaging intracellular H_2_S and cytosolic Ca^2+^. In the vascular tissues of mammals, H_2_S-induced cytosolic Ca^2+^ rise is attributed to Ca^2+^ release from multiple intracellular sources rather than extracellular Ca^2+^ influx [38]. The different regulatory styles of cytosolic Ca^2+^ by H_2_S between plants and mammals would be an interesting topic to be investigated further.

H_2_S regulates various physiological processes by targeting K_ATP_ channels in mammals. H_2_S is an endogenous opener of K_ATP_ channels by interacting with Cys6 and Cys26 in rvSUR1 (Sulphonylurea Receptor 1) subunit of K_ATP_ channel complex through *S*-sulfhydration [47]. In plants, MRP5 (Multidrug Resistance-associated Protein 5) is a homologue of mammalian SUR [48]. In *Arabidopsis*, AtMRP5 not only works as an auxin conjugate transporter in modulating lateral root formation but also acts as a regulator of Ca^2+^ channel in regulating guard cell signaling [49], [50]. MRP5 can be possibly regulated by H_2_S due to the fact that the treatment with Gli (glibenclamide), a typical SUR inhibitor, blocks NaHS-induced stomatal closure [21]. Thus, whether H_2_S regulates Ca^2+^ signaling through the *S*-sulfhydration of MRP5 during lateral root formation needs to be further investigated. In addition, NO may induce Ca^2+^ influx by post-transcriptionally modified Ca^2+^ channel proteins directly [51], [52]. Therefore, it is possible that NO may act parallelly with H_2_S in inducing cytosolic Ca^2+^.

**Figure 6:**
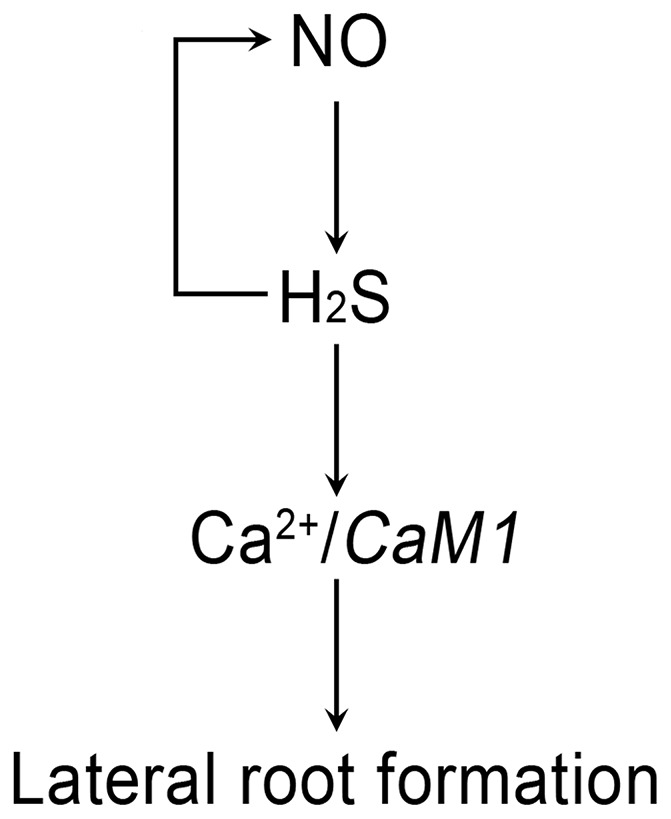
The schematic model for H_2_S regulation of lateral root formation. A positive feedback regulation of NO by H_2_S probably exists in inducing lateral root formation.

The biology of H_2_S in mammals has been significantly advanced, but mining the signaling role of H_2_S in plants is just emerging. Based on our observation, a model could be proposed of the crosstalk between NO and H_2_S in regulating lateral root formation (Figure 6). The current regulatory network involving NO, H_2_S, and Ca^2+^ in regulating later root formation is largely unknown, but our data suggest that H_2_S acting between NO and Ca^2+^ is one of the possible signaling pathway in the complicated network for the regulation of lateral root formation. However, a possible feedback mechanism between NO and H_2_S maybe operating for the induction of lateral root formation. Our present study is the first report of bioimaging endogenous H_2_S in plants, which provides the direct evidence of identifying H_2_S as a true cellular signaling molecule in regulating lateral root formation. These results not only propose a novel component in lateral root signaling but also shed new light on the study of the biological role of H_2_S in plants.
